# Gut microbiota links with cognitive impairment in amyotrophic lateral sclerosis: A multi-omics study

**DOI:** 10.7555/JBR.36.20220198

**Published:** 2022-12-28

**Authors:** Zhenxiang Gong, Li Ba, Jiahui Tang, Yuan Yang, Zehui Li, Mao Liu, Chun Yang, Fengfei Ding, Min Zhang

**Affiliations:** 1 Department of Neurology and Psychiatry, Tongji Hospital, Tongji Medical College, Huazhong University of Science and Technology, Wuhan, Hubei 430030, China; 2 Department of Neurology, SUNY Downstate Medical Center, NY 11226, USA; 3 Department of Anesthesiology and Perioperative Medicine, the First Affiliated Hospital of Nanjing Medical University, Nanjing, Jiangsu 210029, China; 4 Department of Pharmacology, School of Basic Medical Sciences, Fudan University, Shanghai 200032, China

**Keywords:** amyotrophic lateral sclerosis, cognitive decline, gut microbiota, fecal metabolites, bile acids

## Abstract

Recently, cognitive impairments (CI) and behavioral abnormalities in patients with amyotrophic lateral sclerosis (ALS) have been reported. However, the underlying mechanisms have been poorly understood. In the current study, we explored the role of gut microbiota in CI of ALS patients. We collected fecal samples from 35 ALS patients and 35 healthy controls. The cognitive function of the ALS patients was evaluated using the Edinburgh Cognitive and Behavioral ALS Screen. We analyzed these samples by using 16S rRNA gene sequencing as well as both untargeted and targeted (bile acids) metabolite mapping between patients with CI and patients with normal cognition (CN). We found altered gut microbial communities and a lower ratio of *Firmicutes*/*Bacteroidetes* in the CI group, compared with the CN group. In addition, the untargeted metabolite mapping revealed that 26 and 17 metabolites significantly increased and decreased, respectively, in the CI group, compared with the CN group. These metabolites were mapped to the metabolic pathways associated with bile acids. We further found that cholic acid and chenodeoxycholic acid were significantly lower in the CI group than in the CN group. In conclusion, we found that the gut microbiota and its metabolome profile differed between ALS patients with and without CI and that the altered bile acid profile in fecal samples was significantly associated with CI in ALS patients. These results need to be replicated in larger studies in the future.

## Introduction

Amyotrophic lateral sclerosis (ALS) is a fatal neurodegenerative disorder characterized by a progressive degeneration of the upper and lower motor neurons^[1]^. Motor functions of ALS patients would usually deteriorate within three to five years, and they might suffer from respiratory failure in the late stages^[1]^. ALS is devastating to both patients and caregivers due to an absence of curative treatment. Many neuroscientists and neurologists have observed that cognitive and behavioral abnormalities are common among ALS patients^[2]^. Approximately 35% of ALS patients have comorbid cognitive impairments (CI)^[3]^. Cognitive decline severely impairs treatment compliance among patients and causes a burden on caregivers^[4]^, leading to shortened survival spans and dampening the quality of life^[5]^. Cognitive decline in ALS patients primarily manifests with executive, language, and visual space dysfunction^[6]^. A subpopulation of ALS patients could develop behavioral impairments, including loss of interest, apathy, absence of insight, irritability, and aggression^[7–8]^. However, the underlying mechanisms remain unknown.

Among genes related to the pathogenesis of ALS, hexanucleotide GGGGCC repeat expansions in the *C9orf72* gene explain the association between ALS and frontotemporal dementia. About 5%–15% of ALS patients satisfy the diagnostic criteria for frontotemporal dementia^[9]^. However, this *C9orf72* mutation barely occurs in Chinese ALS patients^[10–11]^. Our clinical data suggested that about 38.7% of ALS patients exhibited CI and that 31.5% had behavioral abnormalities^[12]^. Therefore, we proposed an alternative mechanism other than genetic susceptibility that may contribute to the impaired cognition in ALS patients.

Recent studies have elucidated a link between gut microbiota and CI in neurodegenerative disorders, such as Alzheimer's (AD) and Parkinson's disease^[13–14]^. The altered gut microbiota play a role in the CI of patients suffering from neurodegenerative diseases^[15]^. A few microbiomics studies combined with metabolomics have shedded light on cognitive impairment mechanisms in patients with neurodegenerative diseases, including regulating microglial function using secreted metabolites and neurotransmitters^[16–17]^. Although several global study groups have reported altered gut microbiota in ALS patients compared with healthy controls, the conclusions are inconsistent^[18–22]^. To date, no studies have uncovered the mechanistic links between CI and gut microbiota in ALS patients.

In the current study, we first conducted 16S rRNA sequencing of fecal samples from both ALS patients and healthy controls. Then we further compared gut microbiota and untargeted fecal metabolites of the ALS patients with and without CI. Biostatistical analyses revealed that the extracted metabolites significantly differed between the two groups and that these metabolites were mapped to metabolic pathways associated with bile acids. We attempted to identify a link between the gut microbiota and CI in ALS patients, providing clues for future mechanistic studies.

## Materials and methods

### Study design, registrations, and patient consents

We conducted a case-control study of 35 patients with ALS and 35 age- and sex-matched healthy controls and also explored the changes in gut microbiota and fecal metabolites between ALS patients with and without cognitive decline. The current study was approved by the ethics committee of Tongji Hospital, Tongji Medical College, Huazhong University of Science and Technology (TJ-IRB20201219). All participants provided written informed consent, and the study was conducted following the Declaration of Helsinki.

### Subject recruitment and fecal sample collection

**Figure 1:**
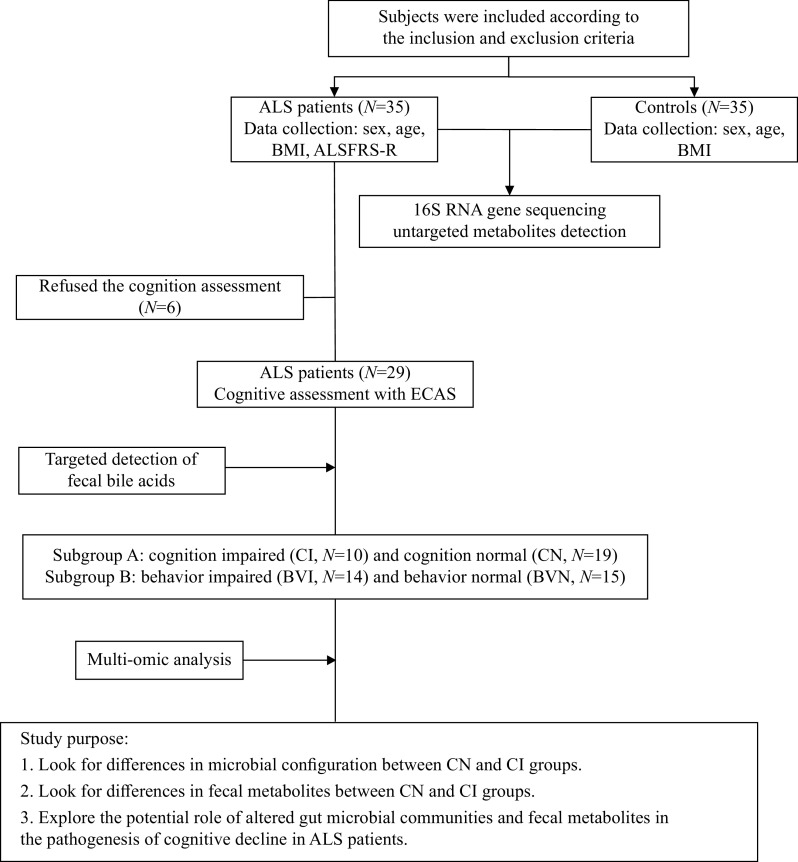
The research flow chart.

The study design is described in ***Fig. 1***. Briefly, 35 ALS patients diagnosed with the revised El Escorial criteria^[23]^ and 35 age- and sex-matched healthy controls were recruited between September 2020 and December 2021. Exclusion criteria were incorporated to exclude diseases with a clear impact on the gut microbiota (***Supplementary Table 1***, available online). Specifically, patients with severe dysphagia were excluded to reduce the biases in nutrient intake. Two fecal samples of each participant were collected from the first bowel moment of the day, and the samples were collected in 15 mL falcon tubes and stored at −80 ℃.

### Assessment of motor function and cognitive function in ALS patients

The Revised ALS Functional Rating Scale (ALSFRS-R) was performed to assess disease severity of ALS patients^[24]^. The Edinburgh Cognitive and Behavioral ALS Screen (ECAS) is specifically designed to identify cognitive and behavioral changes in ALS patients^[6]^. The ECAS evaluates three ALS-specific cognitive domains (language, verbal fluency, and executive function) and two ALS-non-specific cognitive domains (memory and visuospatial function). In the current study, we screened ALS patients with CI and ALS patients with normal cognition (CN), based on the cut-off value of total ECAS scores in Chinese population^[6]^. Additionally, ECAS includes a brief questionnaire to detect behavioral abnormalities, completed by primary caregivers. Patients with an abnormality in at least one behavioral domain were considered to have behavioral impairments (BVI); otherwise, behavior normal (BVN). Two trained investigators performed the ALSFRS-R and the Chinese version of ECAS.

### 16S rRNA gene sequencing and microbiome analysis

The amplicon sequencing of the 16S rRNA gene was conducted by the Shanghai Majorbio Bio-Pharm Technology Co., Ltd. (China), as previously described^[25]^. Briefly, the DNA was extracted (Cat. No. DP712, Tiangen, Beijing, China) and the V3-V4 variable region was amplified with barcode-indexed primers (338F and 806R). Then, amplicons were sequenced using the Illumina MiSeq platform (PE300). The raw sequences were processed as previously described^[25]^. The sequence data has been deposited in the National Genomics Data Center (https://ngdc.cncb.ac.cn/, PRJCA006335). Sobs, Chao, and Ace index were calculated (Mothur, v1.30.2) to evaluate the richness and evenness (α diversity) of the microbial communities^[26–27]^. Principal coordinate analysis (PCoA) and permutational multivariate analysis of variance were used for the comparison of β diversity based on the Bray-Curtis distance^[28]^. The linear discriminant analysis (LDA) of effect size (LEfSe) revealed representative bacterial taxa in each group^[29]^, and the threshold was an LDA score >3.

### Fecal metabolite detection and metabolomic analysis

The Shanghai Majorbio Bio-Pharm Technology Co., Ltd. performed the detection of fecal metabolites. The metabolites were separated using an ExionLC AD System (AB Sciex, Framingham, USA). The Ultra Performance Liquid Chromatography (UPLC) system was coupled with a quadrupole time-of-flight mass spectrometer (AB SCIEX-Triple TOF 5600+, Framingham, USA) and electrospray ionization source. The data acquisition was performed in the Data Dependent Acquisition mode. The metabolites were annotated based on the Human Metabolome Database (HMDB) and Metlin mass spectral database. The orthogonal partial least squares discriminate analysis (OPLS-DA) showed the similarity of fecal metabolites among different groups. Representative metabolites from each group were selected based on the Variable Importance in the Projection (VIP) value in OPLS-DA and the *P*-value within the comparison of concentrations (VIP score >1, *P*<0.05). The metabolites were mapped to biochemical pathways in the Kyoto Encyclopedia of Genes and Genomes (KEGG) for exploring metabolic functions. In the bile acids-targeted detection, the ion fragments were identified in AB SCIEX quantification software OS and manually checked. The concentration of 46 bile acids was evaluated based on the standard curve plotted using the mass spectral peak area as the ordinate and the analytic concentration.

### Statistical analysis

Continuous variables are presented as mean (SD) or median (including 25% quartile and 75% quartile). In comparing the two groups, Student's *t*-test was used for normally distributed data, Mann-Whitney *U*-test was used for non-normally distributed data, and Fisher's exact probability test was employed for categorical data. Spearman's correlation coefficient was calculated in the correlation analysis.* P<*0.05 was considered statistically significant. Statistical analysis was performed using SPSS (version 23.0). Graphs were drawn using R (version 3.4.1) and GraphPad Prism 7.0.

## Results

### Demographic and clinical characteristics

The same number of ALS patients and healthy controls (35 each) were enrolled. No significant difference was observed among age, body mass index (BMI), and sex ratio between the cases and controls (***Table 1***). Among the 35 ALS patients, 32 had a spinal onset, and three had a bulbar onset. The median disease duration in these patients with ALS was 10 months, and the median score of ALSFRS-R was 43.

**Table 1:** Demographic and clinical characteristics

Parameters	Participants				*P*-value^1^	*P*-value^2^
	ALS (*N*=35)	HC *(N*=35)	CN (*N*=19)	CI (*N*=10)		
Demographic information						
Age (years)	54 (8)	54 (9)	50 (6)	59 (10)	0.967^a^	0.030^a^
Female/Male	14/21	14/21	8/11	4/6	1^c^	0.615^c^
BMI (kg/m^2^)	22.15 (2.69)	22.77 (2.11)	22.35 (2.97)	22.02 (3.01)	0.291^a^	0.776^a^
Clinical characteristics						
Duration (months)	10 (6, 24)	–	7 (4, 15)	24 (12, 24)	–	0.006^b^
ALSFRS-R	43 (39, 46)	–	43 (39, 46)	43 (39, 45)	–	0.626^b^
ALSFRS-R, Bulb scores	12 (10, 12)	–	12 (11, 12)	11 (9, 12)	–	0.129^b^
ALSFRS-R, Respiration scores	12 (12, 12)	–	12 (12, 12)	12 (12, 12)	–	0.490^b^
ECAS	–	–	97 (11)	59 (17)	–	-
Data are presented as mean (SD) or median (25% quantile, 75% quantile). *P*-values were calculated by Student's *t*-test^a^, Mann-Whitney *U*-test^b^ or Fisher's exact-test^c^. *P*-value^1^: ALS *vs.* HC; *P*-value^2^: CN *vs.* CI. ALS: amyotrophic lateral sclerosis; HC: healthy control; CN: normal cognition; CI: cognitive impairments; BMI: body mass index; ALSFRS-R: revised ALS Functional Rating Scale; ECAS: the Edinburgh Cognitive and Behavioral ALS Screen; SD: standard deviation.						

### Microbial and metabolomic differences between ALS patients and healthy controls

**Figure 2:**
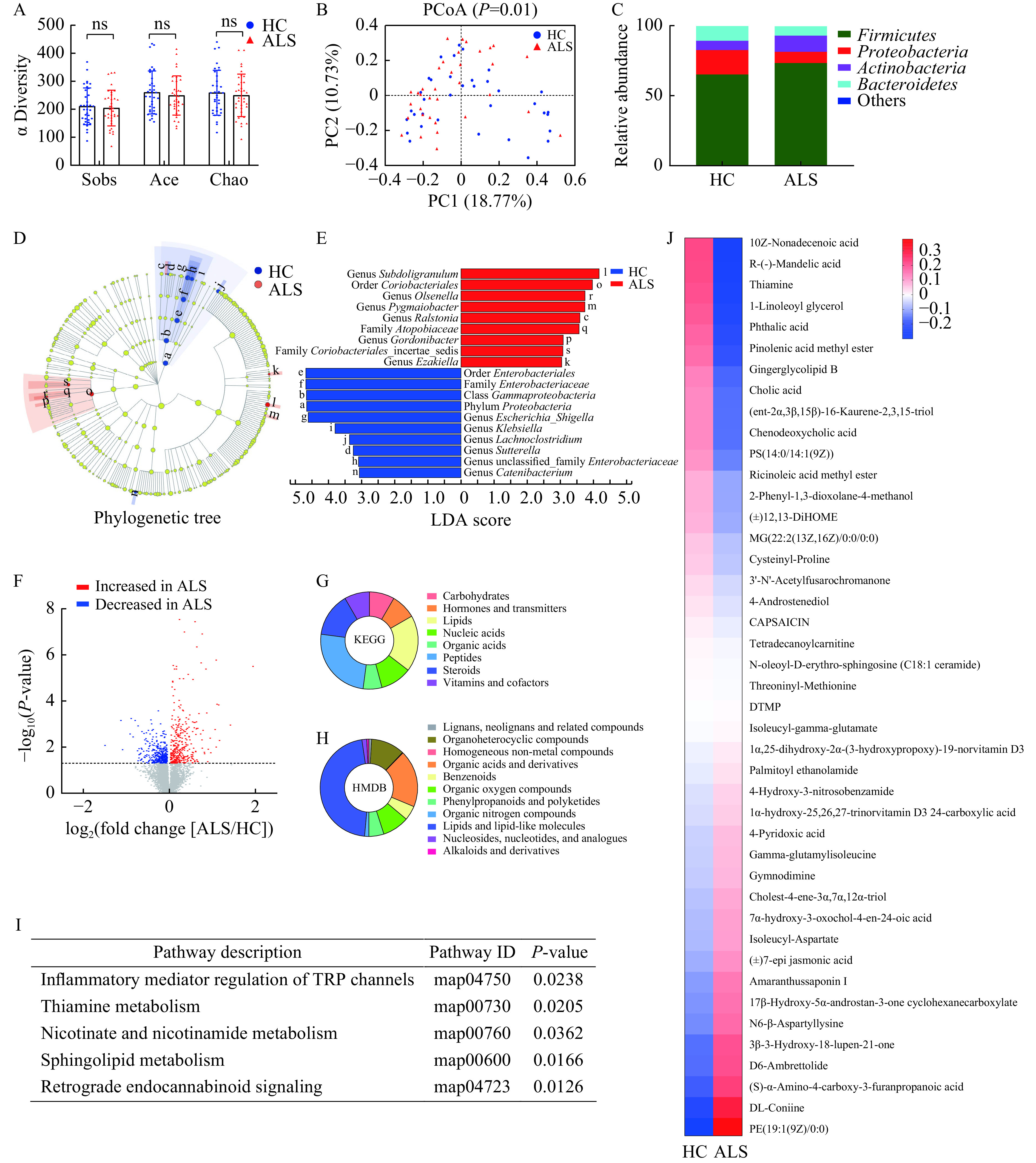
Difference in microbial community and fecal metabolites between ALS patients and healthy controls.

According to the 16S rRNA gene data, there was no difference in the α diversity (*P>*0.05) between ALS patients and healthy controls (***Fig. 2A***). Regarding β diversity, PCoA analysis depicted a significant difference between ALS patients and healthy controls (*P<*0.05) (***Fig. 2B***). Compared with the healthy control group, the phylum *Proteobacteria* significantly decreased in the ALS patient group (***Fig. 2C***). The *Firmicutes* to *Bacteroidetes* (F/B) ratio was comparable between ALS patients and healthy controls (***Supplementary Table 2***, available online). Additionally, the Lefse analysis revealed distinctive bacterial species of each group and the subordination of bacterial species (***Fig. 2D*** and ***E***).

For the untargeted metabolite mapping, 29 ALS patients and 23 healthy controls were included. A total of 14108 metabolites were detected, among which 41 statistically different metabolites were identified from the KEGG and HMDB database (*P<*0.05) (***Fig. 2F***–***H***). Twenty-two metabolites increased, and 19 metabolites decreased, respectively, in the ALS patient group, compared with the healthy control group (***Fig. 2J***). In the KEGG pathway enrichment analysis, those 41 metabolites were mapped to five metabolic pathways (*P<*0.05), including the retrograde endocannabinoid signaling, inflammatory mediator regulation of the transient receptor potential channels, sphingolipid, nicotinamide, and thiamine metabolism (***Fig. 2I***).

### Microbial difference between ALS patients with and without cognitive impairment

**Figure 3:**
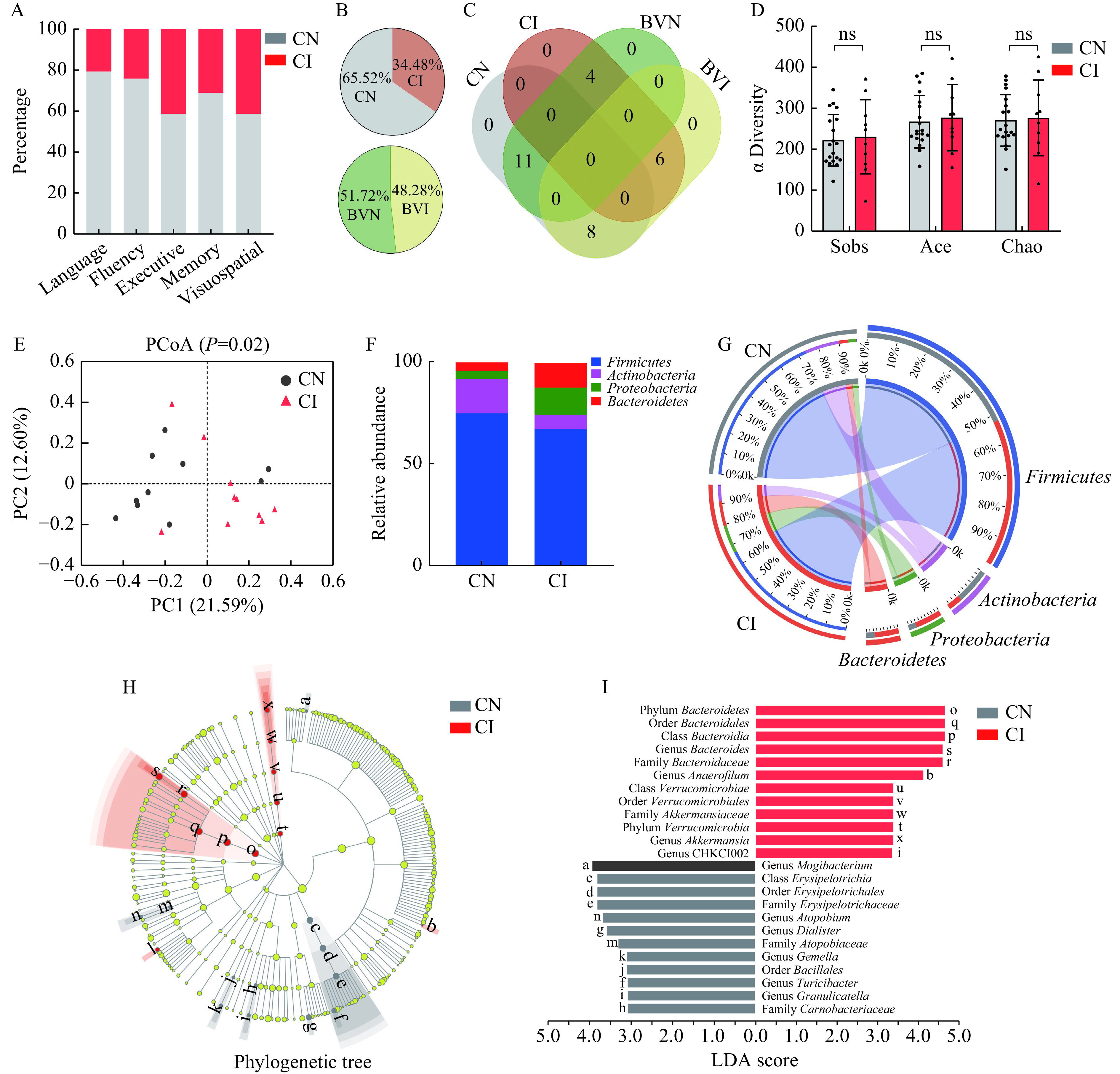
Difference in the microbial community between patients with CN and CI.

Among 29 ALS patients who underwent the ECAS, 10 (34.48%) had CI, and 19 patients (65.52%) had normal cognition (***Fig. 3A*** and ***B***), according to the cut-off score of ECAS in Chinese ALS patients^[6]^. Fourteen patients (48.28%) exhibited at least a single type of BVI (***Fig. 3B***), and six patients showed CI combined with abnormal behaviors (***Fig. 3C***). The microbial α diversity was similar between the CN and CI groups (*P>*0.05) (***Fig. 3D***). The microbial β diversity was significantly different between the CN and CI groups based on the PCoA (*P<*0.05) (***Fig. 3E***). The phylum *Bacteroidetes* (*P<*0.05) and *Proteobacteria* (*P>*0.05) elevated, while *Firmicutes* and *Actinobacteria* reduced in the CI group (*P>*0.05) compared with the CN group (***Fig. 3F*** and ***G***). The F/B ratio was significantly lower in the CI group than that in the CN group (*P<*0.05) (***Supplementary Table 3***, available online). In Lefse analysis, the CI group revealed a higher abundance of bacterial species belonging to *Bacteroidia* and *Verrucomicrobiae* classes, while the CN group showed a higher abundance of bacterial species belonging to the class *Erysipelotrichia* (***Fig. 3H*** and ***I***).

### Fecal metabolite difference between patients with and without CI

**Figure 4:**
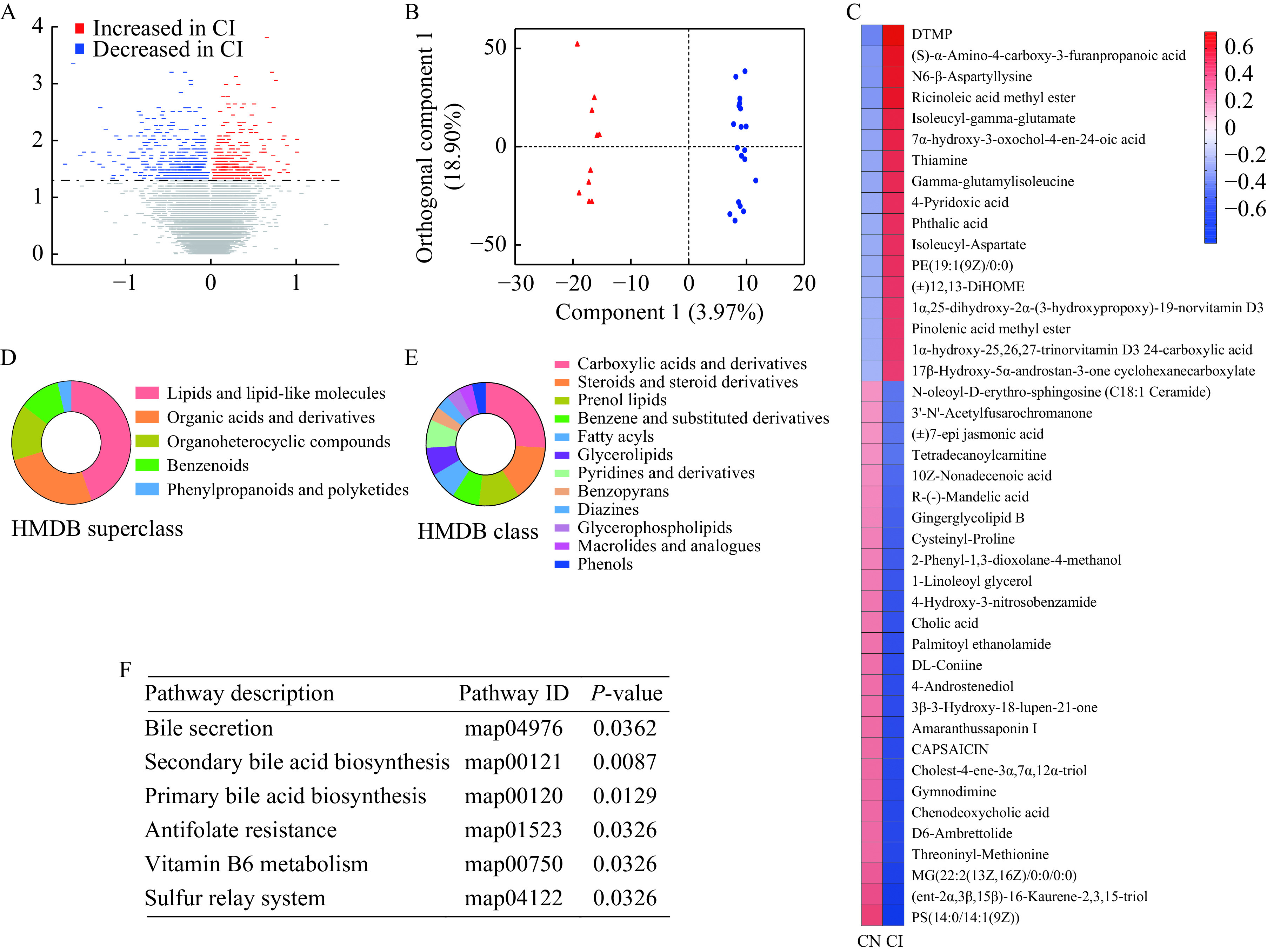
Difference in fecal metabolites between patients with CN and CI.

The OPLS-DA analysis separates the CN and CI groups into discrete distributions, indicating distinctive metabolomic characteristics (***Fig. 4B***). Among 43 metabolites identified in the KEGG and HMDB, 26 metabolites decreased and 17 metabolites increased in the CI group (***Fig. 4C***–***E***), respectively, compared with the CN group. Those 43 metabolites were mapped to six metabolic pathways, including the sulfur relay system, antifolate resistance, vitamin B6 metabolism, bile secretion, primary bile acid biosynthesis, and secondary bile acid biosynthesis (***Fig. 4F***). Patients with CI showed unique metabolic characteristics in fecal samples. Notably, three of six distinctive metabolic pathways were linked to bile acid metabolism.

### Bile acids profile changed in patients with CI

Based on our findings in the untargeted metabolic mapping (***Fig. 4F***), we further undertook targeted bile acids quantification between the CN and CI groups. Primary bile acids (PBAs) include cholic acid (CA), chenodeoxycholic acid (CDCA), and their taurine or glycine bound derivatives. Secondary bile acids (SBAs) were the PBA derivates, including deoxycholic acid (DCA, a CA derivative), lithocholic acid (LCA, a CDCA derivative), ursodeoxycholic acid (UDCA), and α-muricholic acid and their conjugated forms^[30]^. In total, 46 kinds of bile acids were identified. Compared with the CN group, PBAs were significantly lower in the CI group, including CA, CDCA, GlyCA, GlyCDCA, and TaurCDCA (*P<*0.05) (***Table 2*** and ***Supplementary Table 5*** [available online]).

**Table 2:** Statistically different fecal bile acids between CN and CI

Bile acids	Classification	Group		*P*-value
		CN (ng/g)	CI (ng/g)	
Cholic acid	PBA	17476.93 (3392.46, 31117.48)	2298.26 (402.65, 15086.72)	0.035
Chenodeoxycholic acid	PBA	13405.17 (4045.43, 23799.13)	2222.42 (541.38, 13613.2)	0.017
Glycocholic acid	PBA	440.68 (163.06, 1255.82)	99.83 (39.4, 577.28)	0.022
Glycochenodeoxycholic acid	PBA	746.89 (398.69, 1124.03)	218.68 (100.81, 428.34)	0.004
Taurochenodeoxycholic acid	PBA	220.9 (120.53, 407.36)	77.85 (32.10, 223.88)	0.022
Allocholic Acid	SBA	1591.32 (212.42, 2767.59)	152.17 (74.74, 1402.8)	0.039
Alpha-Muricholic acid	SBA	36.88 (19.05)	17.45 (14.45)	0.009
Beta-Muricholic acid	SBA	988.52 (398.5, 2676.31)	265.4 (226.12, 1015.36)	0.048
Chenodeoxycholic Acid 24-Acyl-β-D-glucuronide	SBA	3.14 (1.22, 9.08)	0.39 (0.20, 4.19)	0.039
Glycohyocholic acid	SBA	2.4 (1.36, 5.34)	0.39 (0.34, 1.03)	0.001
Glycoursodeoxycholic acid	SBA	268.64 (106.39, 602.2)	28.84 (23.06, 267.43)	0.008
Lithocholic acid 3-sulfate	SBA	2337.19 (941.51, 8830.24)	461.87 (122.87, 3939.11)	0.035
Norcholic acid	SBA	131.32 (84.62, 299.36)	55.98 (35.71, 157.43)	0.044
Taurohyodeoxycholic acid	SBA	25.91 (10.84, 120.13)	2.44 (0.94, 33.75)	0.013
Tauroursodeoxycholic acid	SBA	24.58 (7.75, 95.88)	2.04 (1.15, 32.88)	0.019
Data are expressed as median (25% quantile, 75% quantile). *P*-values were calculated by Student's t test or Mann-Whitney *U*-test. CN: normal cognition; CI: cognitive impairments; PBA: primary bile acid; SBA: secondary bile acid.				

### The correlation between the altered bile acids and gut microbial species

**Figure 5:**
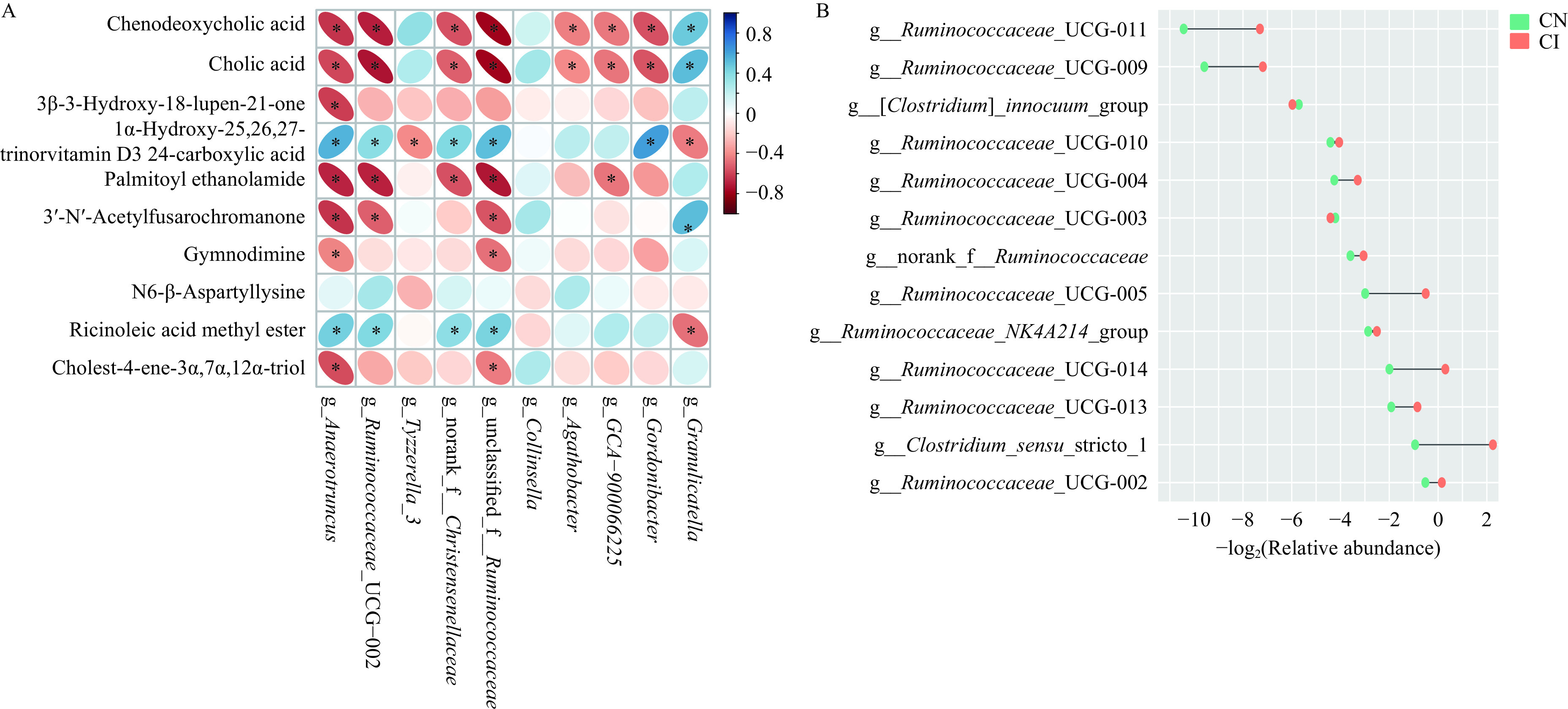
Difference in bacterial species related to bile acids metabolism between patients with CN and CI.

We analyzed the correlations between the abundance of the top 10 microbial genus species and the concentration of the top 10 abundant metabolites. CDCA and CA were associated with eight out of 10 top-expressing genera (***Fig. 5A***). Bacterial species belonging to the family* Ruminococcaceae* and genus *Clostridium* are the known bacteria that play a critical role in transforming PBAs to SBAs, converting CA to DCA, and CDCA to LCA^[31–32]^. Further, we compared the relative abundance of all genera belonging to the family *Ruminococcaceae* between the CN group and the CI group, 10 of 11 genera belonging to the *Ruminococcaceae* family increased in the CI group (***Fig. 5B***).

### Microbial and metabolomic differences between ALS patients with and without behavioral abnormalities

In the current study, 14 ALS patients were divided into patients with behavior impairments (BVI) and 15 ALS patients were divided into patients without behavior impairments (BVN) as described in the methods. There was also no difference in α diversity and β diversity between the BVN and BVI groups (*P*>0.05) (***Supplementary Fig. 1A*** and ***B***, available online). Both the abundance of four dominant phyla and the ratio of F/B were comparable between the BVN and BVI groups (*P*>0.05) (***Supplementary Fig. 1C*** and ***Supplementary Table 4***, available online). Additionally, there was no bacterial species with a LDA score >3 in LEfSe analysis. In comparison of metabolites, there were 22 metabolites with significant differences in concentration between the BVI and BVI groups (***Supplementary Fig. 1D***, available online). However, these metabolites were not significantly mapped to any metabolic pathway. In the quantitative determination of bile acids, only dehydrocholic acid was higher in BVI group compared with the BVN group (***Supplementary Table 6***, available online).

## Discussion

In the current study, we found altered gut microbiota and lowered PBAs levels in fecal samples of ALS patients with CI, compared with patients with normal cognition. Decreased PBAs were associated with increased bacteria that could consume PBAs. The current data suggests that the disruption of gut microbiota-related bile acids metabolism is associated with cognitive decline in ALS.

### Gut microbiota and fecal metabolites differed between ALS patients with and without CI

In the current study, we found evident differences in gut microbiota and fecal metabolites between CN and CI patients, but not between BVN and BVI patients. *Firmicutes* and *Bacteroidetes* belong to bacterial phyla, lower F/B ratio indicates intestinal homeostasis dysregulation^[33]^. We found that the F/B ratio was comparable between ALS patients and healthy controls; however, it was significantly lower in the CI group than that in the CN group. Previously, various gut microbiota studies have been conducted in ALS patients (reviewed in ***Table 3***). In the current study, the changes of gut microbiota between ALS patients and healthy controls have some similarities with those found in previous studies, with a similar α diversity but a different β diversity between ALS patients and healthy controls^[34–38]^. In untargeted metabolic mapping, we found disturbed profiles of lipids and lipid-like molecules in CI patients, including CA CDCA, 1-tetradecanoyl-2-(9Z-tetradecenoyl)-glycero-3-phosphoserine, and 1,3-dihydroxypropan-2-yl (9Z)-tetradec-9-enoate. Particularly, three of six mapped metabolic pathways in KEGG were related to bile acid metabolism, suggesting a potential link between bile acids and cognition in ALS patients. A recent study was conducted with both metagenomics and metabolomics analyses in 10 ALS patients and 10 healthy controls, providing limited conclusive findings^[36]^. In spite of previous studies about the ALS gut microbiota, to the best of our knowledge, we are the first to reveal the differences in gut microbiota and fecal metabolites between patients with and without cognitive decline using a multi-omics approach.

**Table 3:** Literature review of clinical studies on gut microbiota in ALS

References	Subjects (*N*)			Study methods	Main results
	ALS	HC	NDC		
Fang *et al*, 2016^[34]^	6	5	0	High-throughput sequencing (V3–V4 region)	1. The β-diversity was different between ALS and HC. 2. *Dorea* genus increased in ALS, and *Oscillibacter*, *Anaerostripes*, *Lachnospiraceae* genus decreased in ALS.
Rowin *et al*, 2017^[35]^	5	96	0	1. PCR for specific bacterial species 2. Quantitative determination of short chain fatty acids (SCFAs) in fecal samples using mass spectrometry	1. The diversity of the microbiome decreased in ALS (not clearly stated whether α-diversity or β-diversity). 2. A low *Firmicutes*/*Bacteroidetes* (F/B) ratio was found in 3 ALS patients. 3. The level of SCFAs decreased in 1 ALS patient.
Brenner *et al*, 2017^[21]^	25	32	0	454-pyrosequencing (V3–V6 region)	1. Both α-diversity and β-diversity were comparable between ALS and HC. 2. The total number of operational taxonomic units increased in ALS. 3. The relative abundance of the uncultured *Ruminococcaceae* genus was different between ALS and HC.
Zhai *et al*, 2019^[25]^	8	8	0	1. High-throughput sequencing (V4–V5 region) 2. Determination of endotoxin, SCFAs, NO_2_-N/NO_3_-N, and g-aminobutyric acid in fecal samples using enzyme-linked immunosorbent assay (ELISA) kits	1. The F/B ratio and *Methanobrevibacter* genus showed an increased tendency in ALS. 2. *Faecalibacterium* and *Bacteroides* genus (beneficial micro-organisms) decreased in ALS. 3. No significant difference in levels of endotoxin, SCFAs, NO_2_-N/NO_3_-N, and g-aminobutyric acid was found between ALS and HC.
Blacher *et al*, 2019^[20]^	37	29	0	Shotgun sequencing	1. The microbiome composition was different between ALS and HC. 2. Only a marginally significant difference in the abundances of specific bacterial species was found between ALS and HC. 3. ALS microbiomes decreased significantly in the global bacterial gene content related to nicotinamide metabolism.
Zeng *et al*, 2020^[36]^	20	20	0	1. High-throughput sequencing (V4 region) 2. Shotgun sequencing (10 ALS and 10 HC) 3. Untargeted metabolome using liquid chromatography mass spectrometry (LC-MS) (10 ALS and 10 HC)	1. The α-diversity (Shannon index) was different between ALS and HC. 2. *Bacteroidetes* phylum increased in ALS. 3. *Firmicutes* phylum, *Kineothrix*, *Parabacteroides*, *Odoribacter*, *Sporobacter*, *Eisenbergiella*, *Mannheimia*, *Anaerotruncus,* and unclassified *Porphyromonadaceae* genus decreased in ALS. 4. *Enterococcus columbae* positively correlated with 2-(1-ethoxyethoxy) propanoic acid and 3,7-dihydroxy-12-oxocholanoic acid.
Ngo *et al*, 2020^[37]^	49	51	0	High-throughput sequencing (V6–V8 region)	1. The fecal microbiome was not significantly different between ALS and HC. 2. A greater risk for earlier death was reported in ALS patients with increased richness and diversity of the microbiome, and in those with a higher F/B ratio.
Nicholson *et al*, 2020^[38]^	66	61	12	Shotgun sequencing	1. Comparable α-diversity and β-diversity between ALS and HC 2. The relative abundance of the dominant butyrate-producing microbial members decreased in ALS.
Di Gioia *et al*, 2021^[19]^	43	44	0	High-throughput sequencing (V3–V4 region)	1. The α-diversity (Chao1 index) and β-diversity were different between ALS and HC. 2. Microbial members of the *Cyanobacteria* phylum increased in ALS. 3. Microbial members of *Clostridiaceae* family decreased in ALS.
Niccolai, *et al*, 2021^[22]^	19	9	0	1. High-throughput sequencing (V3–V4 region) 2. Determination of 30 kinds of cytokines (test kits) and SCFAs (gas chromatography and mass spectrometry)	1. The F/B ratio decreased in ALS. 2. The relative abundance of butyrate-producing microbial members decreased in ALS. 3. Interleukin-2 (IL-2) and IL-1β increased in ALS. 4. IL-21 decreased in patients with a fast progression.
Note: only the results regarding the fecal microbiome and metabolome were summarized in this table. ALS: amyotrophic lateral sclerosis; HC: healthy control; NDC: neurodegenerative control; PCR: polymerase chain reaction; SCFAs: short chain fatty acids; F/B: *Firmicutes*/*Bacteroidetes*; IL: interleukin.					

### Gut microbiota correlated with cognition by altering fecal bile acids profile in ALS

Based on our findings of altered gut microbiota and in bile acids-related metabolic pathways between CN and CI, we further performed a quantification of fecal bile acids. Decreases in CA, CDCA, and conjugated forms with glycine or taurine and increases in several secondary bile acids were found in the CI group, compared with the CN group. The higher ratios of CA/DCA (CI: 0.63, CN: 0.10) and CDCA/LCA (CI: 0.48, CN: 0.07) demonstrated a higher efficiency of conversion from CA to DCA, CDCA to LCA in the CI group than in the CN group. In humans, PBAs (mainly CA and CDCA) are synthesized in the liver and excreted to the small intestine^[30]^. As PBAs move from the small intestine to the colon, they are converted to SBAs by the biotransformation of the resident microbial community, and the key step is the 7α-dehydroxylation reaction^[32]^. In the colon, nearly 100% of CA and CDCA are converted to DCA and LCA, respectively. However, only a few known bacteria, all from the *Ruminococcaceae* family and *Clostridium* genus, perform the 7α-dehydroxylation^[39]^. Interestingly, 11 genera belonging to the family *Ruminococcaceae* were identified in the current study, and 10 genera increased in the CI group. According to these findings, we propose that the higher efficiency of conversion from PBAs to SBAs in the CI group probably results from alterations in gut microbial communities. However, since 16S sequencing is limited in the annotation of bacteria at the species level^[40]^, we failed to further link the bile acids metabolism to specific bacterial strains. More in-depth basic and clinical studies, such as metagenomic studies and animal intervention studies, need to be further conducted to verify this hypothesis.

In the current study, ALS patients with CI presented with decreased fecal CA and CDCA, leading us to think about whether the impaired cognitive function is potentially linked to altered bile acid metabolic profiles. Bile acids are essential products of cholesterol metabolism, and in addition to playing a key role in lipid metabolism and absorption, recent studies suggest that the bile acid metabolism is associated with cognitive function^[41]^. In bile duct ligation mice, oral administration of obeticholic acid normalized memory function, prevented hippocampal network deficits, and reversed neuronal senescence by activating the farnesoid X receptor^[42]^. Interestingly, CDCA is an endogenous activator of the farnesoid X receptor^[43]^, but whether CDCA can protect cognition by activating the farnesoid X receptor needs to be further explored. It was also reported that oral CDCA supplementation ameliorates neurotoxicity and cognitive deterioration via enhancing insulin signaling in AD model rats^[44]^. In clinical studies, AD patients exhibited significantly lower serum CA and higher DCA levels, compared with healthy controls. The ratio of DCA/CA was significantly correlated with the severity of cognitive decline^[45]^. Elevated DCA was also found in diabetes patients with cognitive impairment^[46]^. Both CA and CDCA could diffuse across the blood-brain barrier, generating aligned concentration in the brain and peripheral tissue^[47]^. Therefore, oral administration of either CA or CDCA could potentially be used as therapeutics for improving cognition. Clinical efficacy of such an approach for slowing cognitive decline in ALS patients could be tested in clinical trials in the future.

### Conclusions

In the current study, we found altered gut microbial communities and bile acid metabolism in ALS patients, highlighting a possible role in the pathogenesis of cognitive decline in ALS patients. To our knowledge, we are the first to reveal connections between gut microbiota and CI in ALS patients. Based on the findings of our multi-omics approach, we propose that novel therapeutics could target bile acid metabolites with the aim of reducing CI in ALS patients. The study could be further strengthened by investigating bile acid metabolites in serum and cerebrospinal fluid, and the current findings need to be verified in studies with a larger sample size, particularly in longitudinal studies that may observe the dynamic changes of gut microbiota and metabolomics overtime.

## SUPPLEMENTARY DATA

Supplementary data to this article can be found online.

## References

[1] (2011). Amyotrophic lateral sclerosis. Lancet.

[2] (2019). Microbiota impacts on chronic inflammation and metabolic syndrome - related cognitive dysfunction. Rev Endocr Metab Disord.

[3] (2018). ALS-specific cognitive and behavior changes associated with advancing disease stage in ALS. Neurology.

[4] (2012). Neurobehavioral dysfunction in ALS has a negative effect on outcome and use of PEG and NIV. Neurology.

[5] (2011). Executive dysfunction is a negative prognostic indicator in patients with ALS without dementia. Neurology.

[6] (2016). The edinburgh cognitive and behavioural ALS screen in a Chinese amyotrophic lateral sclerosis population. PLoS One.

[7] (2017). Screening for cognitive and behavioural impairment in amyotrophic lateral sclerosis: frequency of abnormality and effect on survival. J Neurol Sci.

[8] (2013). Changes in cognition and behaviour in amyotrophic lateral sclerosis: nature of impairment and implications for assessment. Lancet Neurol.

[9] (2018). *C9orf72*-mediated ALS and FTD: multiple pathways to disease. Nat Rev Neurol.

[10] (2017). Genetic epidemiology of amyotrophic lateral sclerosis: a systematic review and meta-analysis. J Neurol, Neurosurg, Psychiatry.

[11] (2015). *C9orf72* hexanucleotide repeat expansions in Chinese sporadic amyotrophic lateral sclerosis. Neurobiol Aging.

[12] (2021). Plasma uric acid helps predict cognitive impairment in patients with amyotrophic lateral sclerosis. Front Neurol.

[13] (2019). Sodium oligomannate therapeutically remodels gut microbiota and suppresses gut bacterial amino acids-shaped neuroinflammation to inhibit Alzheimer's disease progression. Cell Res.

[14] (2021). Regulation of neurotransmitters by the gut microbiota and effects on cognition in neurological disorders. Nutrients.

[15] (2022). Neuroinflammatory remodeling of the anterior cingulate cortex as a key driver of mood disorders in gastrointestinal disease and disorders. Neurosci Biobehav Rev.

[16] (2018). Gut microbiota and their neuroinflammatory implications in Alzheimer's disease. Nutrients.

[17] (2021). Gut microbiota, probiotic interventions, and cognitive function in the elderly: a review of current knowledge. Nutrients.

[18] (2019). Gut microbiota in ALS: possible role in pathogenesis?. Expert Rev Neurother.

[19] (2020). A prospective longitudinal study on the microbiota composition in amyotrophic lateral sclerosis. BMC Med.

[20] (2019). Potential roles of gut microbiome and metabolites in modulating ALS in mice. Nature.

[21] (2018). The fecal microbiome of ALS patients. Neurobiol Aging.

[b22] 22Niccolai E, Di Pilato V, Nannini G, et al. The Gut Microbiota-Immunity Axis in ALS: A Role in Deciphering Disease Heterogeneity?[J]. Biomedicines, 2021, 9(7).

[23] (2000). El Escorial revisited: revised criteria for the diagnosis of amyotrophic lateral sclerosis. Amyotroph Lateral Scler Other Motor Neuron Disord.

[24] (1999). The ALSFRS-R: a revised ALS functional rating scale that incorporates assessments of respiratory function. BDNF ALS Study Group (Phase Ⅲ). J Neurol Sci.

[25] (2019). Intestinal microbiota composition in patients with amyotrophic lateral sclerosis: establishment of bacterial and archaeal communities analyses. Chin Med J.

[26] (2018). Comparison of mothur and QIIME for the analysis of rumen microbiota composition based on 16S rRNA amplicon sequences. Front Microbiol.

[27] (2017). Analysing microbial community composition through amplicon sequencing: from sampling to hypothesis testing. Front Microbiol.

[28] (2020). Persistence of antibiotic resistance genes from river water to tap water in the Yangtze River Delta. Sci Total Environ.

[29] (2011). Metagenomic biomarker discovery and explanation. Genome Biol.

[30] (2006). Bile salt biotransformations by human intestinal bacteria. J Lipid Res.

[31] (2012). Increase in fecal primary bile acids and dysbiosis in patients with diarrhea-predominant irritable bowel syndrome. Neurogastroenterol Motil.

[32] (2017). Interaction of gut microbiota with bile acid metabolism and its influence on disease states. Appl Microbiol Biotechnol.

[33] (2019). What is the healthy gut microbiota composition? A changing ecosystem across age, environment, diet, and diseases. Microorganisms.

[34] (2016). Evaluation of the microbial diversity in amyotrophic lateral sclerosis using high-throughput sequencing. Front Microbiol.

[35] (2017). Gut inflammation and dysbiosis in human motor neuron disease. Physiol Rep.

[36] (2020). The alteration of gut microbiome and metabolism in amyotrophic lateral sclerosis patients. Sci Rep.

[37] (2020). Progression and survival of patients with motor neuron disease relative to their fecal microbiota. Amyotroph Lateral Scler Frontotemporal Degener.

[38] (2021). The human gut microbiota in people with amyotrophic lateral sclerosis. Amyotroph Lateral Scler Frontotemporal Degener.

[39] (2020). Dysbiosis-induced secondary bile acid deficiency promotes intestinal inflammation. Cell Host Microbe.

[40] (2019). Evaluation of 16S rRNA gene sequencing for species and strain-level microbiome analysis. Nat Commun.

[41] (2016). Intestinal crosstalk between bile acids and microbiota and its impact on host metabolism. Cell Metab.

[42] (2022). Anti-cholestatic therapy with obeticholic acid improves short-term memory in bile duct-ligated mice. Am J Pathol.

[43] (2015). Bile acid nuclear receptor FXR and digestive system diseases. Acta Pharm Sin B.

[44] (2019). Chenodeoxycholic acid ameliorates AlCl_3_-induced Alzheimer's disease neurotoxicity and cognitive deterioration via enhanced insulin signaling in rats. Molecules.

[45] (2019). Altered bile acid profile associates with cognitive impairment in Alzheimer's disease-An emerging role for gut microbiome. Alzheimers Dement.

[46] (2010). Metabolic footprint of diabetes: a multiplatform metabolomics study in an epidemiological setting. PLoS One.

[47] (2017). Bile acid signaling pathways from the enterohepatic circulation to the central nervous system. Front Neurosci.

