# Supplementary material for: Gut microbiota links with cognitive impairment in amyotrophic lateral sclerosis: A multi-omics study
Source: J Biomed Res. 2022 Dec 28;37(2):125–37. doi: 10.7555/JBR.36.20220198 (PMC10018415; doi:10.7555/JBR.36.20220198)
Supplement: Supplementary file 1 — Supplementary data to this article can be found online. [file jbr-37-125-S1.pdf]

Available online at www.jbr-pub.org.cn

Open Access at PubMed Central

The Journal of Biomedical Research, 2023 37(1): 1–4

Supplementary Data

JBR

Gut microbiota links with cognitive impairment in amyotrophic lateral sclerosis: a multi-omics study

Zhenxiang Gong[1], Li Ba[1], Jiahui Tang[1], Yuan Yang[1], Zehui Li[1], Mao Liu[3], Chun Yang[4], Fengfei Ding[1,2,✉], Min Zhang[1,✉]

[1]*Department of Neurology and Psychiatry, Tongji Hospital, Tongji Medical College, Huazhong University of Science and Technology, Wuhan, Hubei 430030, China;*
[2]*Department of Pharmacology, School of Basic Medical Sciences, Fudan University, Shanghai 200433, China;*
[3]*Department of Neurology, SUNY Downstate Medical Center, NY 11226, United States;*
[4]*Department of Anesthesiology and Perioperative Medicine, the First Affiliated Hospital of Nanjing Medical University, Nanjing, Jiangsu 210029, China.*

Supplementary Table 1 **Inclusion and exclusion criteria**

Items	Criteria
Inclusion criteria	1. Clinically definite or probable ALS (revised El Escorial criteria)<br>2. 18 < age < 75 years<br>3. 18 < BMI < 28 kg/m²
Exclusion criteria	1. Any possible evidence for familial amyotrophic lateral sclerosis (fALS)<br>2. With other neurodegenerative diseases, such as Alzheimer's disease (AD), Parkinson's disease (PD), Huntington's disease (HD), *etc.*<br>3. With definite gastrointestinal diseases, heart failure, acute infection, tumor, immunodeficiency and autoimmune diseases<br>4. Any use of antibiotics within the last month<br>5. Regular drinking of commercial probiotics within the last year or any drinking of commercial probiotics within the last week<br>6. Obvious symptoms of dysphagia or dyspnea

Supplementary Table 2 **Relative abundance at phylum level between ALS and HC**

Phylum	ALS	HC	*P*-value
Actinobacteria	5.63 (1.66, 13.73)	2.87 (0.87, 5.85)	0.053
Bacteroidetes	2.62 (0.42, 9.65)	3.99 (1.37, 15.57)	0.125
Firmicutes	78.78 (62.53, 86.54)	66.55 (45.64, 81.42)	0.075
Proteobacteria	1.56 (0.87, 10.33)	7.78 (1.22, 33.45)	0.026
Verrucomicrobia	0 (0, 0.01)	0 (0, 0)	0.689
F/B	29.07 (6.47, 138.63)	18.32 (3.72, 64.07)	0.092

Data are presented as median (25% quantile, 75% quantile). *P*-values were calculated by Mann-Whitney *U*-test. ALS: amyotrophic lateral sclerosis; HC: healthy control; F/B: *Firmicutes*/*Bacteroidetes*.

✉Corresponding authors: Fengfei Ding, Department of Pharmacology, School of Basic Medical Sciences; State Key Laboratory of Medical Neurobiology and MOE Frontiers Center for Brain Science, Institutes of Brain Science, Shanghai Medical College, Fudan University, 130 Dong'an Road, Xuhui District, Shanghai 200032, China. Tel: +86-21-54237900, E-mail: fengfei_ding@fudan.edu.cn; Min Zhang, Department of Neurology and Psychiatry, Tongji Hospital, Tongji Medical College, Huazhong University of Science and Technology, 1095 Jiefang Road, Qiaokou District, Wuhan, Hubei 430030, China. Tel: +86-27-83663895, E-mail: zhang_min_3464@126.com.

Received: 07 September 2022; Revised: 05 November 2022; Accepted: 22 November 2022; Published online: 28 December 2022

CLC number: R744.8, Document code: A

The authors reported no conflict of interests.

This is an open access article under the Creative Commons Attribution (CC BY 4.0) license, which permits others to distribute, remix, adapt and build upon this work, for commercial use, provided the original work is properly cited.

© 2023 by the Journal of Biomedical Research.

https://doi.org/10.7555/JBR.36.20220198

Supplementary Table 3 **Relative abundance at phylum level between CN and CI**

Phylum	CN	CI	*P*-value
Actinobacteria	10.88 (5.63, 26.45)	1.9 (1.27, 9.20)	0.066
Bacteroidetes	1.79 (0.21, 8.49)	8.95 (2.35, 16.14)	0.039
Firmicutes	80.69 (62.54, 86.85)	74.68 (52.59, 84.97)	0.383
Proteobacteria	1.29 (0.32, 5.67)	1.51 (1.14, 14.35)	0.215
Verrucomicrobia	0 (0, 0)	0 (0, 43.20)	0.033
F/B	45.55 (8.59, 368.50)	9.10 (3.47, 32.30)	0.022

Data are presented as median (25% quantile, 75% quantile). *P*-values were calculated by Mann-Whitney *U*-test. CN: normal cognition; CI: cognitive impairments; F/B, *Firmicutes*/*Bacteroidetes*.

Supplementary Table 4 **Relative abundance at phylum level between BVN and BVI**

Phylum	BVN	BVI	*P*-value
Actinobacteria	10.92 (3.30, 15.97)	3.90 (1.03, 17.38)	0.106
Bacteroidetes	1.91 (0.15, 9.68)	5.18 (0.61, 13.49)	0.348
Firmicutes	79.04 (53.69, 86.55)	76.47 (64.32, 87.47)	0.485
Proteobacteria	1.24 (0.32, 4.14)	2.49 (0.82, 9.40)	0.256
Verrucomicrobia	0 (0, 0.09)	0 (0, 0)	0.114
F/B	45.40 (6.58, 368,69)	18.79 (5.61, 174.30)	0.458

Data are presented as median (25% quantile, 75% quantile). *P*-values were calculated by Mann-Whitney *U*-test. BVN: behavior normal; BVI: behavior impaired; F/B: *Firmicutes*/*Bacteroidetes*.

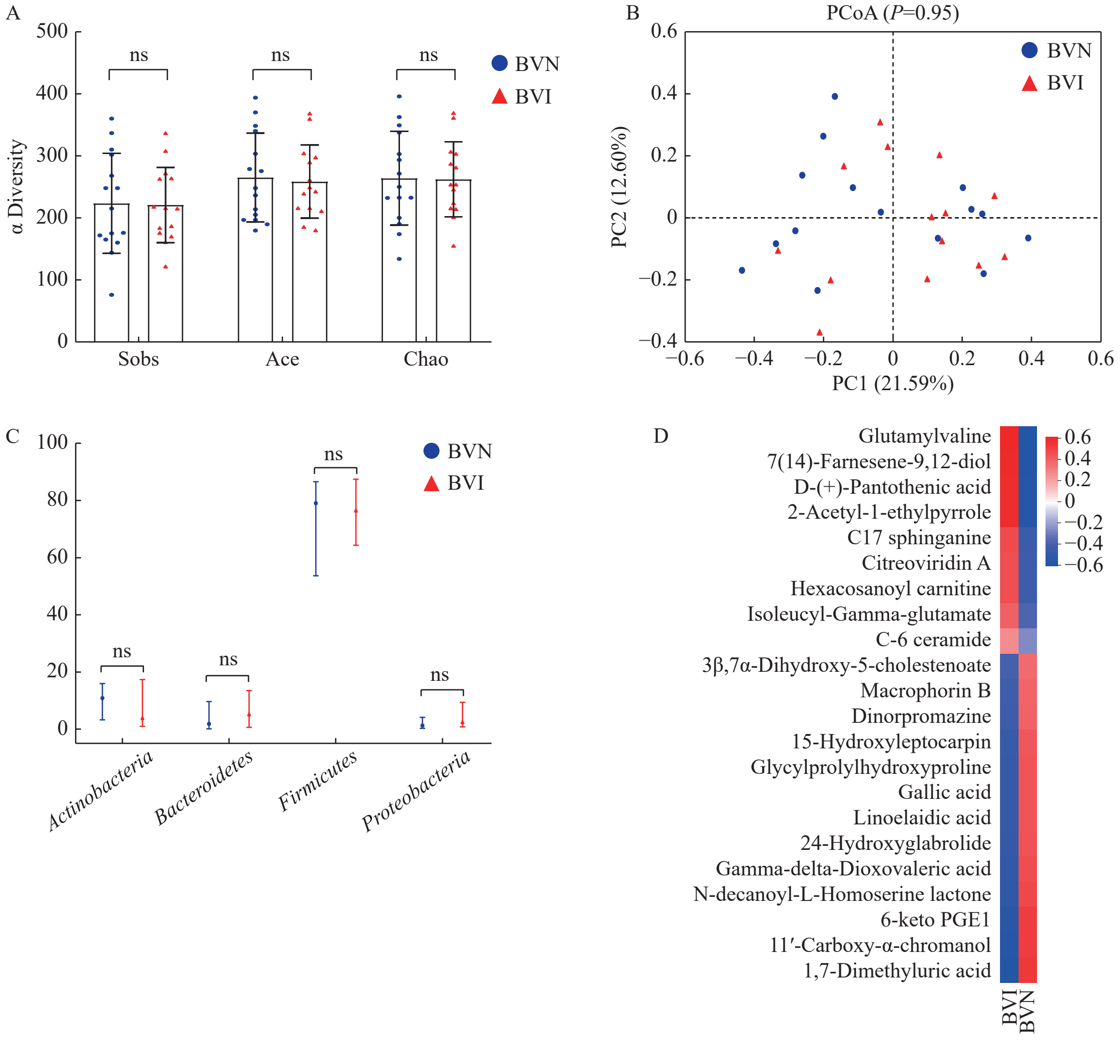

Supplementary Fig. 1 **Difference in microbial community and fecal metabolites between patients with BVN or BVI.** ns: not significant; BVN: behavior normal; BVI: behavior impaired.

Supplementary Table 5 **Comparison of 46 fecal bile acids between CN and CI**

Bile acid	CN (ng/g)	CI (ng/g)	Effect size	*P*-value
Deoxycholic acid	19 303.91 (11 950.51)	16 824.23 (8 588.38)	0.580	0.567
Hyodeoxycholic acid	4 257.27 (2 358.58)	2 947.85 (2 192.8)	1.454	0.157
Alpha-Muricholic acid	36.88 (19.05)	17.45 (14.45)	2.818	0.009
Lithocholic acid	20 577.58 (16 757.16)	22 749.48 (13 842.67)	–0.351	0.728
Isodeoxycholic acid	0.58 (0.26)	0.65 (0.27)	–0.691	0.495
Glycocholic acid	440.68 (163.06, 1 255.82)	99.83 (39.4, 577.28)	–2.294	0.022
Glycochenodeoxycholic acid	746.89 (398.69, 1 124.03)	218.68 (100.81, 428.34)	–2.845	0.004
Taurocholic acid	179.14 (58.02, 800.7)	59.28 (11.74, 232.33)	–1.606	0.108
Taurochenodeoxycholic acid	220.9 (120.53, 407.36)	77.85 (32.10, 223.88)	–2.294	0.022
Glycoursodeoxycholic acid	268.64 (106.39, 602.2)	28.84 (23.06, 267.43)	–2.661	0.008
Allocholic Acid	1 591.32 (212.42, 2 767.59)	152.17 (74.74, 1 402.8)	–2.065	0.039
Ursodeoxycholic acid	6 091.26 (1 900.69, 9 848.19)	2 003.6 (708.65, 7027)	–1.652	0.099
Tauroursodeoxycholic acid	24.58 (7.75, 95.88)	2.04 (1.15, 32.88)	–2.340	0.019
apocholic acid	10.08 (0, 26.02)	8.65 (0, 31.35)	–0.048	0.962
Glycodeoxycholic acid	402.58 (37.63, 790.16)	96.53 (63.79, 486.55)	–1.101	0.271
Glycolithocholic acid	32.33 (2.50, 96.5)	18.96 (3.71, 70.08)	–0.367	0.714
Beta-Muricholic acid	988.52 (398.5, 2 676.31)	265.4 (226.12, 1 015.36)	–1.973	0.048
7-ketoLithocholic acid	3 267.56 (1 118.73, 7 681.85)	992.11 (398.15, 6 928.47)	–1.285	0.199
Tauro-α-muricholic acid	3.1 (1.16, 33.16)	3.33 (1.15, 26.78)	0.000	1.000
Tauro-β-muricholic acid	1.97 (1.27, 13.89)	1.79 (0.73, 5.50)	–0.918	0.359
Omega-Murichoclic acid	519.93 (68.46, 1 856.2)	64.27 (30.5, 545.63)	–1.468	0.142
murideoxycholic acid	168.62 (81.52, 382.15)	153.25 (44.74, 267.45)	–0.826	0.409
Taurohyodeoxycholic acid	25.91 (10.84, 120.13)	2.44 (0.94, 33.75)	–2.478	0.013
Taurohyocholic acid	1.04 (0.48, 3.39)	1.04 (0.28, 2.30)	–0.596	0.551
taurolithocholic acid	16.58 (4.34, 23.81)	8.56 (1.47, 12.04)	–1.422	0.155
Taurodeoxycholate acid	38.45 (26.23, 175.27)	18.72 (1.4, 47.88)	–1.606	0.108
Cholic acid	17 476.93 (3 392.46, 31 117.48)	2 298.26 (402.65, 15 086.72)	–2.111	0.035
Chenodeoxycholic acid	13 405.17 (4 045.43, 23 799.13)	2 222.42 (541.38, 13 613.2)	–2.386	0.017
hyocholic acid	1 466.61 (361.96, 2 853.8)	624.34 (157, 1 510.36)	–1.331	0.183
norcholic acid	131.32 (84.62, 299.36)	55.98 (35.71, 157.43)	–2.019	0.044
Glycohyocholic acid	2.4 (1.36, 5.34)	0.39 (0.34, 1.03)	–3.349	0.001
23-Nordeoxycholic acid	9.41 (0.55, 26.8)	15.46 (0.80, 30.39)	–0.322	0.747
isolithocholic acid	14 864.25 (231.85, 26 994.75)	22 587.64 (9 516.01, 37 160.78)	–0.918	0.359
12-ketolithocholic acid	54.94 (38.6, 131.63)	70.61 (40.55, 126.74)	–0.184	0.854
dehydrolithocholic acid	956.96 (131.08, 4 816.54)	3 270.94 (2 033.57, 6 014.95)	–0.780	0.435
Lithocholic acid 3-sulfate	2 337.19 (941.51, 8 830.24)	461.87 (122.87, 3 939.11)	–2.111	0.035
Chenodeoxycholic acid-3-β-D-glucuronide	99.43 (65.40, 343)	86.85 (50.45, 137.36)	–1.055	0.291
3β-Ursodeoxycholic acid	2 322.27 (546.43, 4 058.25)	1 046.1 (165.36, 4 834.84)	–0.780	0.435
3-Dehydrocholic acid	472.57 (114.00, 4 138.09)	102.71 (12.98, 792.39)	–1.927	0.054
Chenodeoxycholic Acid 24-Acyl-β-D-glucuronide	3.14 (1.22, 9.08)	0.39 (0.20, 4.19)	–2.065	0.039
12-ketochenodeoxycholicacid	1 768.2 (369.24, 16 349.26)	406.47 (109.68, 1 929.66)	–1.698	0.090
7,12-Diketolithocholic acid	80.62 (25.98, 221.79)	26.79 (8.34, 62.82)	–1.927	0.054
Dehydrocholic acid	2.27 (1.38, 3.62)	1.85 (1.04, 4.53)	–0.849	0.396
Ursocholic acid	10 507.16 (1 377.77, 23 380.96)	1 094.41 (202.85, 9 942.72)	–1.514	0.130
7-ketodeoxycholic acid	2 642.46 (1 795.27, 17 525.9)	1 216.89 (338.84, 9 486.95)	–1.331	0.183
3β-Cholic acid	4 237.48 (769.19, 9 479.69)	1 037.88 (137.85, 5 457.19)	–1.331	0.183

Data are presented as median (25% quantile, 75% quantile). *P*-values were calculated by Mann-Whitney *U*-test. CN: normal cognition; CI: cognitive impairments.

Supplementary Table 6 **Comparison of 46 fecal bile acids between BVN and BVI**

Bile acid	BVN	BVI	Effect size	*P*-value
Ursodeoxycholic acid	6 199.46 (4 564.13)	4 524.07 (3 635.19)	1.088	0.286
Deoxycholic acid	17 531.73 (11 921.98)	19 431.47 (9 840.22)	–0.466	0.645
Hyodeoxycholic acid	4 253.34 (2 547.17)	3 326.18 (2 104.63)	1.064	0.297
Alpha-Muricholic acid	29.53 (19.37)	30.89 (20.79)	–0.183	0.856
Dehydrocholic acid	1.9 (1.02)	3.8 (3.03)	–2.233	0.040
Glycocholic acid	424.7 (146.61, 897.75)	297.86 (84.73, 747.25)	–0.611	0.541
Glycochenodeoxycholic acid	496.08 (391.04, 1 124.03)	383.57 (179.72, 1 089.59)	–0.786	0.432
Taurocholic acid	325.08 (24.96, 787.05)	80 (37.63, 351.23)	–0.655	0.513
Taurochenodeoxycholic acid	220.9 (103.44, 585.21)	101.48 (70.35, 281.97)	–1.047	0.295
Glycoursodeoxycholic acid	268.09 (45.77, 383.54)	151.62 (28.09, 561.74)	–0.349	0.727
Allocholic Acid	1 215.52 (119.89, 2 767.59)	639.79 (104.84, 1 875.25)	–0.742	0.458
Tauroursodeoxycholic acid	24.58 (3.08, 95.88)	16.4 (2.12, 29.85)	–0.480	0.631
apocholic acid	10.08 (0, 26.02)	8.65 (0, 31.16)	0.000	1.000
Glycodeoxycholic acid	331.64 (37.63, 734.99)	346.07 (73.91, 783.53)	–0.262	0.793
Glycolithocholic acid	9.44 (1.99, 96.5)	35.95 (5.96, 70.84)	–0.742	0.458
Beta-Muricholic acid	733.54 (254.82, 2 676.31)	680.26 (263.99, 1 071.89)	–0.262	0.793
7-ketoLithocholic acid	5 575.11 (616.49, 7 681.85)	2 602.89 (830, 4 803.18)	–0.524	0.600
Tauro-α-muricholic acid	3.1 (1.16, 46.42)	3.78 (1.35, 15.59)	–0.175	0.861
Tauro-β-muricholic acid	1.97 (1.18, 13.89)	2.11 (0.97, 5.17)	–0.349	0.727
Omega-Murichoclic acid	426.57 (30.57, 2 071.08)	250.69 (45.24, 672.18)	–0.436	0.663
murideoxycholic acid	183.13 (56.95, 382.15)	152.46 (71.67, 332.42)	–0.393	0.694
Taurohyodeoxycholic acid	29.2 (4.40, 120.13)	15.94 (2.81, 28.57)	–0.698	0.485
Taurohyocholic acid	1.05 (0.57, 1.8)	0.85 (0.29, 3.07)	–0.436	0.663
taurolithocholic acid	8.25 (1.69, 16.77)	12.12 (5.69, 33.38)	–1.528	0.127
Taurodeoxycholate acid	29.66 (8.54, 62.64)	44.53 (14.14, 182.28)	–0.742	0.458
Lithocholic acid	14 529.43 (1 612.12, 38 816.5)	27 715.41 (18 640, 30 730.43)	–0.786	0.432
Cholic acid	25 852.42 (903.55, 31 117.48)	7 784.96 (977.27, 18 338.82)	–1.004	0.315
Chenodeoxycholic acid	18 067.76 (2 562.88, 23 246.58)	4 818.78 (1 966.83, 15 016.37)	–0.960	0.337
hyocholic acid	446.78 (253.43, 2 558.18)	1 157.54 (569.66, 3 218.12)	–0.960	0.337
norcholic acid	151.76 (57.89, 224.69)	89.85 (47.67, 178.17)	–0.873	0.383
Glycohyocholic acid	1.49 (0.68, 3.03)	1.29 (0.40, 4.61)	–0.044	0.965
23-Nordeoxycholic acid	15.51 (0.55, 27.53)	9.64 (3.65, 26.11)	–0.175	0.861
isolithocholic acid	4 499.56 (231.85, 37 278.71)	20 873.81 (10 639.70, 28 829.47)	–0.742	0.458
12-ketolithocholic acid	53.38 (38.60, 112.34)	73.75 (40.63, 138.57)	–0.567	0.570
dehydrolithocholic acid	460.18 (34.53, 4 816.54)	2 913.2 (842.86, 5 523.13)	–0.786	0.432
Lithocholic acid 3-sulfate	1 982.18 (642.94, 7 149.78)	1 547.74 (365.88, 6 979.24)	–0.349	0.727
Chenodeoxycholic acid-3-β-D-glucuronide	97.7 (55.50, 239.3)	96.95 (50.45, 376.51)	–0.175	0.861
3β-Ursodeoxycholic acid	1 875.87 (439.07, 4 315.57)	1 733.41 (451.54, 4 076.74)	–0.131	0.896
3-Dehydrocholic acid	712.52 (37.96, 4 138.09)	199.85 (58.99, 850.56)	–0.524	0.600
Chenodeoxycholic Acid 24-Acyl-β-D-glucuronide	3.74 (0.43, 31.85)	0.89 (0.38, 3.51)	–1.528	0.127
12-ketochenodeoxycholicacid	1 671.12 (107.12, 16 349.26)	893.87 (322.93, 3 059.98)	–0.044	0.965
7,12-Diketolithocholic acid	37.05 (16.77, 126.75)	57.79 (22.29, 221.36)	–0.524	0.600
Ursocholic acid	7 879.37 (242.21, 30 668.33)	6 522.81 (1 344.95, 12 188.63)	–0.175	0.861
7-ketodeoxycholic acid	6 695.79 (265.09, 17 525.9)	2 459.53 (667.74, 6 547.47)	–0.480	0.631
Isodeoxycholic acid	0.54 (0.40, 0.85)	0.56 (0.46, 0.71)	–0.524	0.600
3β-Cholic acid	1 811.27 (143.74, 9 635.12)	2 260.49 (316.05, 5 541.74)	–0.044	0.965

Data are presented as median (25% quantile, 75% quantile). *P*-values were calculated by Mann-Whitney *U*-test. BVN: behavior normal; BVI: behavior impaired.
